# Psychiatric Comorbidities and Liver Injury Are Associated With Unbalanced Plasma Bile Acid Profile During Methamphetamine Withdrawal

**DOI:** 10.3389/fendo.2021.801686

**Published:** 2022-01-03

**Authors:** Yuru Ma, Hongjin Wu, Huawei Wang, Fengrong Chen, Zhenrong Xie, Zunyue Zhang, Qingyan Peng, Jiqing Yang, Yong Zhou, Cheng Chen, Minghui Chen, Yongjin Zhang, Juehua Yu, Kunhua Wang

**Affiliations:** ^1^ National Health Commission (NHC) Key Laboratory of Drug Addiction Medicine (Kunming Medical University), First Affiliated Hospital of Kunming Medical University, Kunming, China; ^2^ Centre for Experimental Studies and Research, First Affiliated Hospital of Kunming Medical University, Kunming, China; ^3^ Medical School, Kunming University of Science and Technology, Kunming, China; ^4^ Yunnan University, Kunming, China

**Keywords:** methamphetamine withdrawal, bile acid, psychiatric comorbidities, liver injury, crosstalk

## Abstract

**Background:**

The pathogenesis of methamphetamine usedisorders (MUDs) remains largely unknown; however, bile acids may play arole as potential mediators of liver injury and psychiatric comorbidities.The aim of this study was to characterize bile acid (BA) profiles in plasmaof patients with MUDs undergoing withdrawal.

**Methods:**

Liver functions and psychiatric symptoms wereevaluated in a retrospective cohort (30 MUDs versus 30 control subjects) andan exploratory cohort (30 MUDs including 10 subjects each at the 7-day,3-month, and 12-month withdrawal stages versus 10 control subjects). BAcompositions in plasma samples from MUD patients in the exploratory cohortwere determined by gas-liquid chromatography.

**Results:**

Both psychiatric comorbidities andmethamphetamine-induced liver injury were observed in patients in both MUDcohorts. The plasma concentrations of the total BA, cholic acid (CA), andchenodeoxycholic acid (CDCA) were lower in MUD patients relative tocontrols. The maximum decline was observed at the 3-month stage, withgradual recovery at the 12-month stage. Notably, the ratios of deoxycholicacid (DCA)/CA and lithocholic acid (LCA)/CDCA were statistically significantat the 3-month stage comparing with controls. Significant correlations werefound between the LCA/CDCA and taurolithocholic acid (TLCA)/CDCA ratios andthe levels of alanine transaminase and aspartate aminotransferase, andbetween the LCA/CDCA ratio and the HAM-A score.

**Conclusion:**

BA profile during METH withdrawal weremarkedly altered, with these unbalanced BAs being associated with liverinjury. The associations between BA profiles and psychiatric symptomssuggest an association between specific BAs and disease progression,possibly through the liver-brain axis.

## Introduction

Methamphetamine (METH), a potent addictive psychostimulant, wasinitially developed from its parent drug amphetamine, but the prevalence of METH abuse has been significantly increasing (1).According to the 2019 State Council of China report, approximately 56.1% ofregistered drug abusers suffered from some form of METH dependence (The State Council China, 2018 China Drug Situation Report, 2019).

Anxiety and depression, the most common psychiatric symptoms ofpatients with methamphetamine use disorders (MUDs), may persist for 6 months orlonger during its withdrawal (2), and may evenrecur and persist throughout life (1, 3). Our previous cross-sectional study revealedimportant associations between the key neurotransmitters GABA, serotonin andcholine, and the severities of anxiety and depression symptoms in MUDs (4, 5). Despite studies showing that METH is neurotoxic, pharmacologic interventions focusedon modulating monoaminergic pathways have largely failed and no medications to datehave been approved by the U.S. Food and Drug Administration (FDA) for treating METHdependence (6).

In addition to the stimulating and psychotropic effects to the brain(7), METH damages multiple peripheralorgans (8), including the liver, intestines,kidneys, and muscles, of which the liver is the most vulnerable organ (9, 10). For instance, patients with MUDs have shown serological evidence of METH-inducedacute liver injury and chronic liver diseases, and animal models of MUDs have shownhistological evidence of acute hepatotoxicity and oxidative stress (10, 11). Furthermore, associations among METH-induced liver injury, increased peripheral andbrain ammonia, and long-term depletions of dopamine and serotonin have been observedin METH-treated rat models (12–14), suggesting that liver injury may beassociated with the process of neurological impairment in patients with MUDs (11, 15). Thus, these systematic impairments including acute or chronic liver injury mayinterfere with pharmacologic interventions targeting brain dysfunction, leading topoor outcome. To date, however, the underlying molecular mechanisms involving theseassociations among peripheral organs and the CNS have not been extensively investigated.

Bile acids (BAs) are a large group of structurally related moleculesderived from cholesterol and synthesized exclusively in the liver (16). Although BAs were thought to primarilyfunction to expedite the digestion and absorption of dietary lipids and lipophilicvitamins by forming micelles in the small intestine (17), they could function to signal through receptors on various celltypes throughout the body, including the CNS and other organ systems (18). Abnormal circulating bile acid metabolitelevels in the patients with Alzheimer’s disease predicted worse outcomes(19). Alterations in cholesterol and BAmetabolism have been shown to contribute to the development of neurodegenerative andneurological diseases, such as Alzheimer’s disease, Parkinson’s disease,and multiple sclerosis (20–22). Recent reports of gut-based bariatricsurgery suggest that chronically elevate systemic BA concentrations and attenuatecocaine-induced cumulative dopamine increase, and this surgery reducesreward-related behavior and psychomotor sensitization to cocaine in a mouse model (23). Moreover, fecal BA excretion levelhas been shown to correlate with alcohol abuse and abstinence (24). Thus, we hypothesized that peripheral BA profiles mayserve as a potential diagnostic biomarker or therapeutic target for substance usedisorders (SUDs). However, the direct role of BAs in the context of METH dependenceand withdrawal remains unclear.

The present study was designed to investigate thedynamic pattern of BAs in patients with MUDs currently undergoing METH withdrawaland to analyze the correlations among anxiety and depression scales,neurotransmitters, laboratory parameters associated with liver and kidney function, glycolipid metabolism, and BAs. These findings might uncover the crosstalk betweenthe liver and the central nervous system (CNS) and provide clues toward a betterunderstanding of the role of BAs in MUDs.

## Materials and Methods

### Participant Cohorts

The present study recruited two independent cohorts from a jointprogram of drug detoxification and rehabilitation in the First AffiliatedHospital of Kunming Medical University and the Kunming Drug RehabilitationCenter between July 2017 and October 2019. The retrospective cohort consisted of30 male MUDs undergoing withdrawal and 30 male healthy control subjects (HCs).The exploratory cohort consisted of 30 male MUDs undergoing withdrawal, 10 eachat the 7-day, 3-month, and 12-month withdrawal stages, and 10 age-matched HCs.Subjects were excluded if they had any other Diagnostic and Statistical Manualof Mental Disorders-5 (DSM-5) axis I or II disorders, other than amphetaminedependence; were positive for anti-HIV or anti-HCV antibodies; had anyneurological disorders or serious medical conditions; or were multi-substanceabusers including abuse of an opioid containing acetaminophen, which can causeliver damage.

All protocols and recruitment procedures wereapproved by the Research Ethics Committee of the First Affiliated Hospital ofKunming Medical University (2018-L-42), and all participants provided writteninformed consent before enrollment.

### Scale Administration

Interviews with a professionally trainedinterviewer were conducted simultaneously with the collection of biologicalsamples. The HAM-A scale consists of 14 questions, seven elements examining psychological stress and seven examining physical stresses (25). Total scores of < 17, 18~24,25~30, and >30, indicated mild, mild to moderate, moderate to severe, andsevere grades of stress severity, respectively. The HAM-D scale consists of a24-item questionnaire that measures the severity of depressive symptoms, withtotal scores > 20 considered indicative of major depression (26).

### Blood Tests

Fasting blood specimens were collected from studyparticipants into 10 mL EDTA-2Na vacuum tubes, and the blood samples werecentrifuged at 1,500 g for 15 min. The plasma was transferred to a new tube andcentrifuged at 20,000 g at 4°C for 15 min. Thesupernatants were aliquoted and stored at -80°C until analysis. Biochemicalparameters were measured using the Beckman Coulter Synchron DxC800 ChemistryAnalyzer (Beckman, USA). Concentrations of neurotransmitters were measured byultra-performance liquid chromatography coupled to tandem mass spectrometry(UPLC-MS/MS; ACQUITY UPLC-Xevo TQ-S, Waters Corp., Milford, MA, USA).

### Targeted Metabolomics Analysis of BAs

Samples were prepared and BA concentrations measured as described (27). Briefly, an internal standardsolution containing six internal standards was added to each plasma sample orstandard solution and centrifuged. The internal standard solution contained 100nM concentrations of d4-glycocholic acid (GCA), d4-taurocholic acid (TCA),d4-cholic acid (CA), d4-glycodeoxycholic acid (GDCA), and d4-deoxycholic acid(DCA) and a 200 nM concentration of d4-lithocholic acid (LCA).

BAs were analyzed using a Waters ACQUITY ultraperformance LC system coupled with a Waters XEVO TQ-S mass spectrometer with anESI source controlled by MassLynx 4.1 software. Chromatographic separations wereperformed on an ACQUITY BEH C18 column (1.7 μM, 100 mm × 2.1 mminternal dimensions) (Waters). Raw UPLC-MS data obtained in negative mode wereanalyzed using Target Lynx applications manager version 4.1 (Waters) to obtaincalibration equations and determine the concentrations of each BA in these samples (28).

### Statistical Analysis

Demographic, clinical, and biochemical parameterswere compared using SPSS Statistics version 23.0 software (IBM Corp., Armonk,NY, USA). Categorical variables using Chi-squared tests. If each group ofcontinuous variables satisfied both the normality test (Shapiro-Wilk normalitytest) and the homogeneity of variance test (Levene’s test), t-tests wereused to conduct variance analysis between two groups whereas one-way ANOVA wereused to conduct variance analysis among three groups; Else if any group ofcontinuous variables could not satisfy the Shapiro-Wilk normality test or theLevene’s test, Wilcoxon rank-sum Test were used to conduct varianceanalysis between two groups whereas Kruskal-Wallis test were used to conductvariance analysis among three groups. All p values were corrected by Bonferronimethod, an adjusted-p value <0.05 was considered statistically significant.Box plots and correlations analyses were performed and visualized the usingpheatmap, ggplot2 and ggpubr packages in R (v 3.6.3).

## Results

### Characteristics of the Study Participants

The clinical characteristics of the retrospective cohort(Research cohort 1) are shown in **Table 1**. Sixty men, 30 MUDs and 30 HCs, aged25–50 years were enrolled. The duration of METH use in the 30 MUDs rangedfrom 61 to 107 months. The major routes of METH administration were smoking andnasal insufflation. There were no significant differences in age, body massindex (BMI), level of education, and self-reported annual income between thesetwo groups (**Table 1**).

**Table 1:** Characteristics of study participants from the retrospective cohort.

	MUDs	HCs	*p_.adj_*
NO.	30	30	NA
Age,years	37.17 ± 4.32	35.28 ± 6.54	0.67
BMI,kg/m2	22.16 ± 1.28	23.47 ± 0.98	0.36
METH abuse history, months	68.79 ± 21.23	NA	NA
Education	13/10/6/1	11/10/7/2	0.21
Income	3/12/6/9/0	3/12/6/6/3	0.14
HAM-A	11.47 ± 2.71	8.30 ± 2.71	2.47×10^-4^
HAM-D	11.83 ± 3.93	8.60 ± 3.93	4.91×10^-3^
Total serum protein	87.27 ± 5.16	82.89 ± 4.69	1.09×10^-3^
ALB, g/L	55.71 ± 3.85	53.33 ± 2.63	7.03×10^-3^
GLB, g/L	37.2 ± 3.28	33.42 ± 3.81	1.23×10^-4^
ALB/GLB	2.25 ± 0.61	1.97 ± 0.53	0.06
ALT, IU/L	33.31 ± 7.70	24.73 ± 7.18	3.78×10^-5^
AST, IU/L	33.97 ± 5.52	25.32 ± 10.94	2.85×10^-4^
AST/ALT	1.20 ± 0.39	1.00 ± 0.21	0.01
TBIL, umol/L	11.60 ± 3.04	10.22 ± 1.32	0.03
PAB, g/L	318.37 ± 55.86	293.60 ± 47.40	0.07
Urea, mmol/L	4.10 ± 1.12	4.75 ± 1.12	0.03
Cr, umol/L	84.65 ± 6.61	84.90 ± 5.67	0.88
UA, umol/L	369.88 ± 46.74	369.88 ± 39.13	1.00
CHOL, mmol/L	6.18 ± 1.16	6.12 ± 0.73	0.81
TG, mmol/L	2.20 ± 1.06	1.63 ± 0.88	0.03
HDL, mmol/L	3.26 ± 1.23	2.33 ± 1.03	2.48×10^-3^
LDL, mmol/L	1.99 ± 0.91	2.61 ± 0.88	0.01

Data are mean ± SD. P values were adjusted with Bonferroni method. Education levels: illiteracy/primary school/middleschool/college; Income levels: monthly0~1000¥/1000~3000¥/3000~5000¥/5000~10000¥/10000+¥.HAM-A, Hamilton Rating Scale for Anxiety; HAM-D, Hamilton Depression Rating Scale; ALB, Albumin; GLB, Globulin; ALT, Alaninetransaminase; AST, Aspartate aminotransferase; TBIL, Serum totalbilirubin; PAB, Prealbumin; Cr, Creatinine; UA, Uric Acid; CHOL, cholesterol; TG, triacylglycerol; HDL, high-density lipoprotein; LDL, low-density lipoprotein; NA, not available.

To determine the dynamic alterations inpsychiatric symptoms and liver damage during METH withdrawal, a second,prospective cohort (Research cohort 2) was recruited. The demographiccharacteristics of this cohort have been previously described (4, 5) and are shown in **Table S1**. There were no significant differences in age, BMI,METH-use history, and education level among the three MUD subgroups and betweenthe MUD and HC groups. Although self-reported annual income was not wellbalanced, this was corrected in the subsequent statistical analyses.

### Co-Existence of Liver Injury and Psychiatric Symptoms in MUDs

Questionnaires assessing the HAM-A and HAM-D rating scales, whichwere developed to quantify the severity of anxiety and depression, respectively,were administered by experienced interviewers. Compared with the HCs, the MUDsin Research cohort 1 had significantly higher scores on both the HAM-A (p =2.47×10^-4^) and HAM-D (p = 4.91×10^-3^)scales. In addition, measurements of liver injury-related blood parameters (**Table 1**) showedthat plasma concentrations of alanine transaminase (ALT, p =3.78×10^-5^) and aspartate aminotransferase (AST, p =2.85×10^-4^) were significantly higher in the MUD than in theHC group. These findings indicate that the MUDs in Research cohort 1 had obviouspsychiatric comorbidities and METH-induced liver damage.

**Figure 1:**
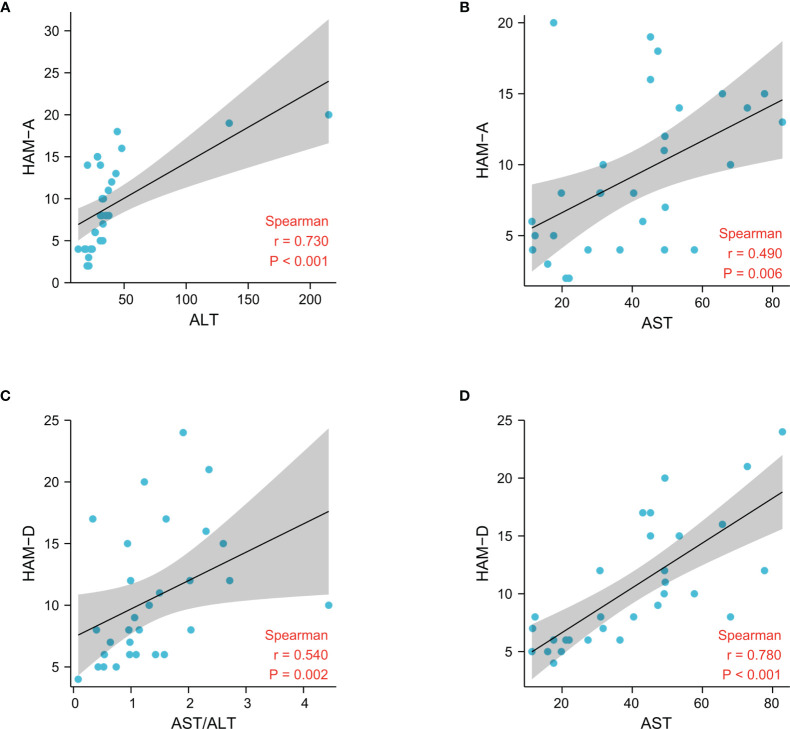
Associations between liver damage and psychiatric comorbidities after METH withdrawal in the patient cohorts, as determined by Spearman correlation analysis.

The psychiatric symptoms and blood concentrationsof peripheral neurotransmitters in Research cohort 2 have been reported (5). Blood parameters associated with glucoseand lipid metabolism, as well as liver and kidney function, were also assessed.Both ALT and AST levels were significantly higher in the MUDs than in the HCs (**Table S1**), consistent with the findings in Research cohort 1.Interestingly, most of the parameters differing in these two groups, includingAST, ALT, and triglyceride (TG) concentrations, as well as scores on the HAM-Aand HAM-D scales, showed greatest significance in the 10 MUD patients at the3-month withdrawal stage. HAM-A scores correlated significantly with theconcentrations of ALT (p < 0.001, r = 0.73), AST (p = 0.006, r = 0.49), TG (p< 0.001, r = 0.58) and LDL-cholesterol (p = 0.01, r = 0.47), whereas HAM-Dscores correlated significantly with the concentrations of AST (p < 0.001, r= 0.78), TG (p = 0.004, r = 0.51) and HDL-cholesterol (p = 0.004, r = -0.52),and the AST/ALT ratio (p = 0.002, r = 0.54). Taken together, these findingsshowed that liver and psychiatric symptoms were altered and correlated with eachother in patients undergoing their first year of METH withdrawal (**Figure 1** and **Figure S1**).

### Dynamics of Plasma BA Concentrations During METH Withdrawal

BA has been reported to play critical roles in neurodegenerativeand neurological diseases (5, 29). To determine the dynamic changes in BAconcentrations at various stages of withdrawal, 42 BAs were simultaneously evaluated in both MUDs and HCs from Research cohort 2 (**Table S2**).Intergroup differential analysis showed significant alterations in plasma BAs ofall three MUD subgroups, with similar trends for BA profile and blood parametersassociated with liver and psychiatric symptoms. These variations were greater atthe 3-month withdrawal stage than at the 7-day and 12- month stages. Forexample, the total BA concentration in plasma was significantly lower in all 30MUDs than in the HCs (p = 8.66×10^-3^). The maximum reductionoccurred at the 3-month stage (p = 5.21×10^-3^), with theseconcentrations gradually recovering at the 12-month stage (p = 0.59). Theprimary BAs, including CA and CDCA, were significantly lower in all three MUDsubgroups than in the HCs (p_CA_ = 7.44×10^-5^ &p_CDCA_ = 9.79×10^-3^), with the greatest reductionsoccurring at 3-months (p_CA_ = 1.08×10^-5^ &p_CDCA_ = 4.27×10^-3^). The concentrations ofsecondary BAs, including HCA and UDCA, were significantly lower in all MUDs thanin the HCs (p_HCA_ = 0.01 & p_UDCA_ = 0.02), with only besignificantly lower at the 3-month stage in the three MUD subgroups(p_HCA_ = 3.05×10^-3^ & p_UDCA_ =4.13×10^-3^). The concentrations of conjugated BAs such asGCDCA, TCDCA and GCA, were not changed in all MUDs relative to the HCs, while the significance was only observed at the 3-month stage (p_GCDCA_ =0.03, p_TCDCA_ = 0.04, p_GCA_ = 0.04).

**Figure 2:**
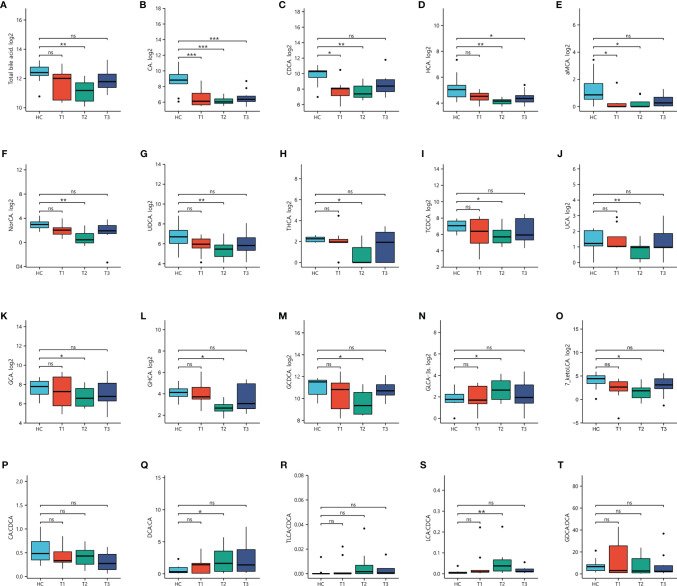
Concentrations of bile acids in patients undergoing METH withdrawal and in healthy controls (HCs). Statistical significance were detected in three stages of METH withdrawal compared to HCs. *p < 0.05; **p < 0.01; ***p < 0.001 for between group comparisons. ns, no significance.

Assessments showed that 11 BAs, including four secondary BAs(hyocholic acid (HCA), a-muricholic acid (aMCA), 23-norcholic acid (NorCA), andursodeoxycholic acid (UDCA)), and seven conjugated BAs (THCA,taurochenodeoxycholic acid (TCDCA), ursocholic acid (UCA), glycohyocholic acid(GHCA), glycocholic acid (GCA), glycochenodeoxycholic acid (GCDCA), andGLCA-3S), were significantly altered only in MUD patients at the 3-monthwithdrawal stage (**Table S2** and **Figure 2**), suggestingthat alterations in key BAs in MUDs during withdrawal were stage specific.

In addition, the ratios of selected BAs were calculated toinvestigate the enzymatic activities involved in BA metabolism. The CA/CDCAratio was used to determine the shifts in BA synthesis from the primary to thealternative pathway; the ratios of secondary to primary BAs (DCA/CA andLCA/CDCA) were used to determine the changes in enzymatic activity towardshifted production of secondary BAs; and the GDCA/DCA and TLCA/DCA ratios wereused to determine whether the dysregulation in secondary BAs was correlated withtaurine or glycine conjugation (19). Interestingly, the ratios of CA/CDCA and DCA/CA were differed significantly inthe MUD and HC groups, whereas the other ratios did not differ. Comparisons ofthe three MUD subgroups with the HC group showed that the DCA/CA and LCA/CDCAratios differed significantly only at the 3-month stage. These results suggest that changes in BA metabolism, particularly in the production of secondary BAs,were stage specific in patients undergoing METH withdrawal.

**Figure 3:**
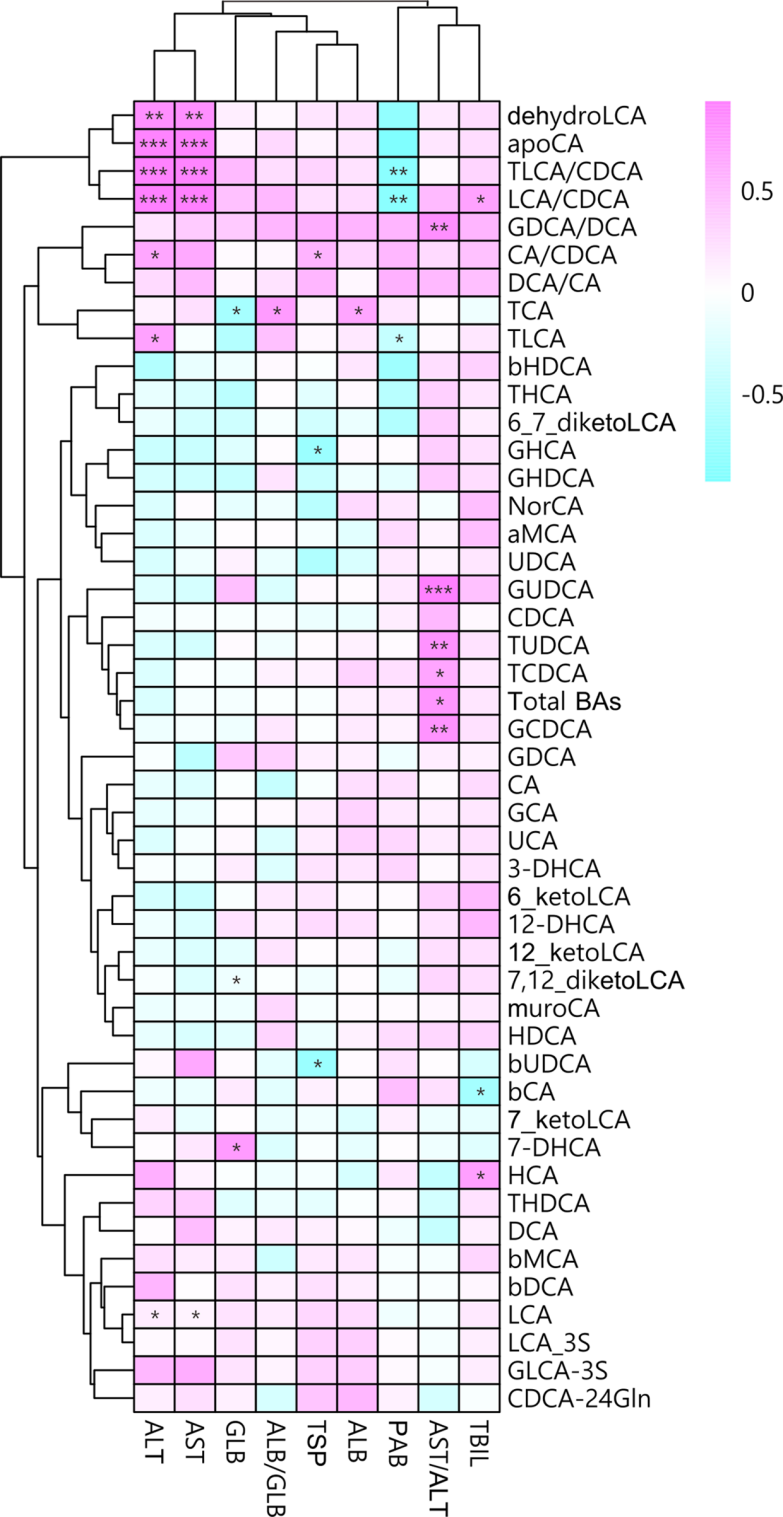
Associations between plasma bile acid concentrations andliver damage after METH withdrawal, as determined by Spearman correlation analysis. Red indicates positive correlations and blue indicates negative correlations, with darker colors indicating stronger correlations. *p < 0.05; **p < 0.01; ***p < 0.001 for between group comparisons.

To further determine the associations betweenplasma BAs and liver injury in MUDs, the Spearman correlations between BAprofiles and plasma concentrations of ALT and AST were calculated and analyzed.The LCA/CDCA ratio correlated significantly correlated with both ALT (r = 0.93,p = 5.03×10^-4^) and AST (r = 0.93, p =1.37×10^-4^) concentrations (**Figure 3**). Similarly, the TLCA/CDCA ratiocorrelated significantly with ALT (r = 0.91, p = 2.26×10^-4^) andAST (r = 0.90, p = 4.33×10^-4^) concentrations. These resultsindicate that BA profiles during withdrawal are markedly altered, and that theseunbalanced BAs are associated with liver injury in patients with MUDs.

### Unbalanced Plasma BAs Were Associated With Psychiatric Comorbidities and Neurotransmitters During METH Withdrawal

**Figure 4:**
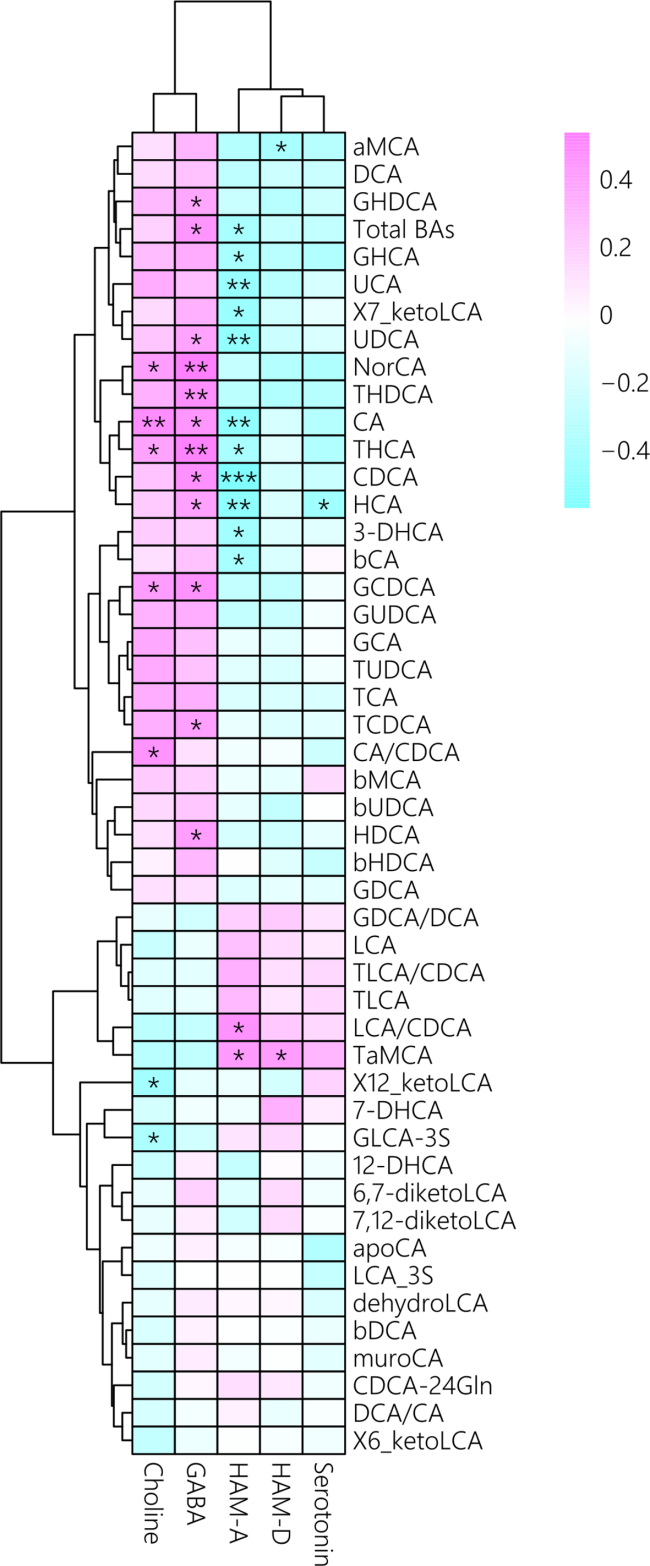
Associations between plasma bile acid concentrations with psychiatric comorbidities and neurotransmitter concentrations after METH withdrawal, as determined by Spearman correlation analysis. Red indicates positive correlations and blue indicates negative correlations, with darker colors indicating stronger correlations.*p < 0.05; **p < 0.01; ***p < 0.001 for between group comparisons.

We also investigated the correlations among BA concentrations,psychiatric comorbidities and neurotransmitter concentrations. Theconcentrations of total BAs, bCA, CA, CDCA, UCA, THCA, GHCA, HCA, UDCA,7_ketoLCA and 3-DHCA were negatively correlated with HAM-A score in MUDs (**Figure 4**), of which theCDCA and HAM-A score showing the strongest negative correlation (r = -0.57, p =0.001). In contrast, the LCA/CDCA ratios (r = 0.46, p = 0.01) and TaMCA (r =0.42, p = 0.02) correlated positively with HAM-A scores.

In addition, CA/CDCA ratios, as well as theconcentrations of CA, GCDCA, NorCA, and THCA, were positively correlated withcholine concentrations, whereas the concentrations of total BAs, CA, CDCA,GCDCA, GHDCA, HCA, THCA, HDCA, NorCA, TCDCA, THDCA, and UCA were positively correlated with GABA concentrations (**Figure 4**). Interestingly, serotonin concentration correlated only with HCA concentration. Altogether, these results indicated that unbalanced BA profiles were associated with psychiatric symptomsand altered neurotransmitters in MUD patients during METH withdrawal.

## Discussion

This study recruited two cohorts of patients with MUDs andage-matched HCs and assessed the concentrations of their circulating BAs,neurotransmitters, and blood parameters related to liver and kidney function andglycolipid metabolism. To our knowledge, this study is the first to show that BAprofiles were significantly altered in patients with MUDs, and that these unbalancedBAs were associated with both liver injury and psychiatric comorbidities in patient sduring the first year of METH withdrawal.

Mood problems (depression and anxiety) and cognitive impairments havebeen associated with liver damage, although these associations were investigatedprimarily in patients with non-alcoholic fatty liver disease (NAFLD) andnon-alcoholic steatohepatitis (NASH) (30). More recently, epidemiological studies have shown that patients with psychosis areat greater risk of presenting with damaged liver function (31), and are at increased risk of chronic liver diseases (32), even during early stages of psychosis (33). Studies using animal modelssuggested that liver dysfunction could reduce METH clearance, increase brain drugconcentrations, and therefore enhance its psychotropic effects on locomotor activityin a dose-dependent manner (34).Hyperthermia-dependent liver damage has been observed in mice after acuteadministration of METH, accompanied by increased plasma aspartate, ALT, and plasmaammonia concentrations (12). Theco-occurrence of liver injury and psychiatric comorbidities was validated in twoindependent cohorts, with ALT and AST concentrations being associated with theseverity of symptoms of anxiety and depression. Although it is unclear whether liverinjury persists along with long-lasting psychiatric comorbidities in MUDs yearsafter withdrawal, this study provides evidence supporting the critical role ofcrosstalk between dysregulation of the liver-brain axis and psychiatric symptomsduring the first year of METH withdrawal.

BAs are the end-products of cholesterol metabolism and are mainlyinvolved in liver, biliary, and intestinal diseases (35, 36). Most primary BAs areproduced in the liver, and can be modified, by conjugation with glycine or taurine,and stored in the gall bladder until they are secreted into gut, where they arefurther modified by enzymes present in gut bacteria to produce secondary BAs (37). During this process, most BAs arereabsorbed and undergo enterohepatic recirculation through the portal venous system,with only a small fraction reaching the systemic circulation. Even though, theselection of plasma BAs could be used as diagnostic biomarkers to distinguishpatients with schizophrenia (38) or diabetes (39) from healthy controls. To elucidatethe role of BAs in the liver-brain axis in MUDs, this study profiled plasma BAs,finding that the concentrations of several key BAs were significantly lower in MUDpatients than in HCs. These BA deficiencies during METH withdrawal were consistentwith psychiatric comorbidities observed in MUDs, providing further evidence that BAshave neuroprotective functions in neurodegenerative diseases (40). The total BA concentration was found to correlatenegatively with both HAM-A and HAM-D scales, but positively correlated with theconcentrations of the neurotransmitters, choline and GABA, suggesting thatexcessively low total BA was associated with worse psychiatric comorbidities. Thistrend was observed for other differential BAs, including CA, CDCA, THCA, HCA, UCA,UDCA, NorCA and GCDCA, although these differences did not reach statisticalsignificance. These results suggest that BA deficiency is an important feature forpatients undergoing METH withdrawal and that BA deficiency may influence neuronalfunctions and enhance the risk of dementia (41). In addition, serum and fecal BA concentrations were shown todecrease in patients of irritable bowel syndrome with constipation (42). Because irritable bowel syndrome involvesinteractions between the intestines and brain and because the constipation phenotypeis very common in MUD patients and animal models of MUD (43, 44), these twoconditions may share some pathophysiological processes, with BA-mediated signalingpathways playing important roles in linking peripheral non-neuronal organ systemswith the CNS (21).

The exact mechanisms underlying the roles of BAs in the liver and CNSare not fully understood. Utilizing mass spectrometry-based targeted metabolomicstechnology and statistical analysis, we measured five selected BA ratios, findingthat the production of secondary BAs was markedly altered in patients undergoingMETH withdrawal, and that the CA/CDCA, TLCA/CDCA and LCA/CDCA ratios were associatedwith ALT and AST concentrations. Mechanistically, altered BA profiles may reflectimbalances in lipid metabolism during METH withdrawal. Hyperlipidemia, especiallyexcess TGs, has been reported to affect the development of neural cognition and mooddisorders through putative mechanisms such as brain blood barrier dysfunction or animbalance in amyloid metabolism (45–47). The present studyshowed that the plasma concentrations of TGs and LDL-cholesterol correlatedpositively with both HAM-A and HAM-D scores, whereas the plasma concentrations ofHDL-cholesterol correlated negatively with HAM-D scores. These findings suggestedthat hyperlipidemia was associated with worse psychiatric comorbidities in patientsduring METH withdrawal.

Altered production of secondary BAs in patients with MUDs mayindicate a significant change their gut microbiota. This hypothesis is supported bystudies showing that alterations in gut microbiota play key roles in the regulationof host metabolism and therefore contribute to severe withdrawal symptoms (48, 49). In addition, altered circulating BAs may affect CNS function by activating FXR inneurons and TGR5 in glial cells, thereby modulating neuroinflammatory andneuropsychiatric behaviors (50). However,this hypothesis has not been experimentally confirmed in patients with MUDs.

This study had several strengths. First, the characteristics ofpatients with MUDs in both cohorts were systematically analyzed. Second, theexploratory cohort included patients at different stages of METH withdrawal, withfindings in these subgroups showing that the critical importance of the time windowfor potential clinical intervention. Third, this study explored clues to peripheralsystems other than the CNS, which are relatively easy to obtain and to be developedas biomarkers. Finally, this study provided in-human evidence that excessively lowBA concentrations and imbalances are adverse factors for psychiatric comorbiditiesand liver injury in METH withdrawal.

This study, however, also had several limitations. First, the samplesizes were relatively small. A study with relative larger sample size shouldhypothetically obtain more accurate results. Second, this was an observational studyshowing statistical associations; therefore, causality could not be determined.Third, because the gut microbiota has been shown to affect the neuropsychiatricbehaviors associated with substance withdrawal (49, 51), future studies shouldinvestigate the association between the composition of gut microbiota and themechanisms of BA metabolism during METH withdrawal.

In conclusion, the present study evaluated the plasmaBA profile in patients with MUDs, finding that deficiencies in overall BAs and inthe production of secondary BAs were associated with psychiatric symptoms as well asMETH-induced liver injury. Additional studies are needed to determine the molecularmechanisms underlying the crosstalk between the liver and the CNS.

## Data Availability Statement

The original contributions presented in the study areincluded in the article/**Supplementary Material**. Further inquiries can be directed to the corresponding authors.

## Ethics Statement

The studies involving human participants were reviewed and approved by the Research Ethics Committee of the First Affiliated Hospital of Kunming Medical University. The patients/participants provided their written informed consent to participate in this study.

## Author Contributions

JHY and KW designed the study and supervised the project. YM, HJW, HWW, FC, ZX, ZZ, QP, JQY, YZ, CC, MC, and YJZ collected the data. JHY, YM, and HJW did the data analysis and interpretation. JHY took the lead in writing the manuscript. All authors reviewed the report and approved the final version.

## Funding

This work was supported by grants from the Yunnan Fundamental Research Projects (Grant No. 202101AU070114), Science and Technology Department of Yunnan Province (Grant No. 2019FB096, 202001AV070010, 202002AA100007), and Academic leader project for health commission of Yunnan Province (Grant No. D-2017007), and Yunling Scholar (Grant No. YLXL20170002).

## Conflict of Interest

The authors declare that the research was conducted in the absence of any commercial or financial relationships that could be construed as a potential conflict of interest.

## Publisher’s Note

All claims expressed in this article are solely those of the authors and do not necessarily represent those of their affiliated organizations, or those of the publisher, the editors and the reviewers. Any product that may be evaluated in this article, or claim that may be made by its manufacturer, is not guaranteed or endorsed by the publisher.

## Supplementary Material

The Supplementary Material for this article can befound online at: https://www.frontiersin.org/articles/10.3389/fendo.2021.801686/full#supplementary-material

Supplementary Figure 1 Associations of dyslipidemia parameters with psychiatric comorbidities **(A–D)** and bile acid concentrations **(E–H)** after METH withdrawal, as determined by Spearman correlation analysis.
