# Supplementary material for: Psychiatric Comorbidities and Liver Injury Are Associated With Unbalanced Plasma Bile Acid Profile During Methamphetamine Withdrawal
Source: Front Endocrinol (Lausanne). 2022 Jan 3;12:801686. doi: 10.3389/fendo.2021.801686 (PMC8761939; doi:10.3389/fendo.2021.801686)
Supplement: Supplementary Figure 1 — Associations of dyslipidemia parameters with psychiatric comorbidities (A–D) and bile acid concentrations (E–H) after METH withdrawal, as determined by Spearman correlation analysis. [file DataSheet_1.zip › 2Revised Supplementary Material Presentation/2revised_supplementary Tables.docx]

Table S1: Characteristics of study participants from the exploratory cohort.

|  | HCs(n=10) | T1 (n=10) | T2 (n=10) | T3 (n=10) | Control vs T1 | | Control vs T2 | | Control vs T3 | |
| --- | --- | --- | --- | --- | --- | --- | --- | --- | --- | --- |
|  | mean | | | | *p* | *p_.adj_* | *p* | *p_.adj_* | *p* | *p_.adj_* |
| Age,years | 36.71±7.32 | 36.07±7.77 | 36.93±7.81 | 36.73±8.38 | 0.91 | 0.91 | 0.85 | 0.91 | 0.91 | 0.91 |
| BMI,kg/m2 | 21.36±1.68 | 21.56±1.69 | 21.70±1.33 | 21.42±1.52 | 0.85 | 0.97 | 0.76 | 0.97 | 0.76 | 0.97 |
| METH abuse history, months | NA | 7.4±2.25 | 7.34±3.35 | 7.63±2.93 | NA | NA | NA | NA | NA | NA |
| Education | 3/3/3/1 | 3/3/3/1 | 3/3/4/0 | 3/5/2/0 | 0.65 | 1.00 | 0.29 | 1.00 | 0.82 | 1.00 |
| Income** | 0/3/4/3/0 | 1/4/2/3/0 | 1/4/2/2/1 | 3/3/1/2/1 | 0.85 | 1.00 | 0.77 | 1.00 | 0.25 | 1.001 |
| HAM-A | 4.9±2.42 | NA | 13.6±3.86 | 9±5.31 | NA | NA | 6.00×10^-4^ | 1.90×10^-3^ | 0.04 | 0.04 |
| HAM-D | 8.2±3.82 | NA | 15.2±5.33 | 11.7±6.36 | NA | NA | 5.00×10^-3^ | 0.02 | 0.21 | 0.21 |
| Choline | 3741.7±1409 | NA | 1597.7±889 | 2797.9±1043 | NA | NA | 7.00×10^-4^ | 1.20×10^-3^ | 0.17 | 0.25 |
| GABA | 30.5±7.5 | NA | 10.6±5.1 | 23.9±4.6 | NA | NA | 1.34×10^-6^ | 9.58×10^-6^ | 0.05 | 0.06 |
| Serotonin | 44.7±44.3 | NA | 162±152.4 | 84.5±51 | NA | NA | 0.03 | 0.33 | 0.61 | 0.88 |
| Total serum protein | 82.62±1.01 | 88.76±1.67 | 91.75±1.11 | 88.61±1.99 | 3.50×10^-3^ | 0.03 | 1.07×10^-7^ | 9.64×10^-7^ | 4.40×10^-3^ | 0.04 |
| ALB, g/L | 52.41±0.51 | 54.14±1.08 | 54.95±0.81 | 54.23±0.84 | 0.08 | 0.15 | 0.01 | 0.01 | 0.07 | 0.08 |
| GLB, g/L | 30.2±0.77 | 34.61±1.08 | 36.8±1.03 | 34.38±1.34 | 4.40×10^-3^ | 0.02 | 4.75×10^-7^ | 2.14×10^-6^ | 4.50×10^-3^ | 0.02 |
| ALB/GLB | 1.73±0.06 | 1.59±0.05 | 1.52±0.05 | 1.59±0.05 | 0.17 | 0.26 | 3.30×10^-3^ | 5.90×10^-3^ | 0.12 | 0.12 |
| ALT, IU/L | 27.45±1.59 | 40.19±7.83 | 34.83±5.96 | 50.25±18.42 | 0.02 | 0.05 | 0.08 | 0.09 | 0.02 | 0.03 |
| AST, IU/L | 28.52±3.73 | 37.13±7.43 | 47.34±10.92 | 69.74±30.12 | 0.21 | 0.27 | 0.02 | 0.03 | 0.01 | 0.03 |
| AST/ALT | 1.35±0.08 | 1.03±0.45 | 1.56±0.8 | 1.17±0.79 | 0.50 | 0.50 | 2.00×10^-3^ | 4.50×10^-3^ | 0.03 | 0.04 |
| TBIL, umol/L | 11.5±1.22 | 7.27±1.59 | 6.17±0.56 | 7.1±1.05 | 0.08 | 0.17 | 1.00×10^-4^ | 3.00×10^-4^ | 0.02 | 0.04 |
| PAB, g/L | 313±7.88 | 295.89±24.53 | 332.63±14.53 | 276.9±24.57 | 0.29 | 0.32 | 0.17 | 0.17 | 0.04 | 0.04 |
| Urea, mmol/L | 4.75±1.37 | 3.3±0.66 | 3.71±0.65 | 4.6±1.94 | 4.36×10^-5^ | 3.22×10^-4^ | 2.34×10^-3^ | 0.02 | 0.78 | 0.89 |
| Cr, μmol/L | 81.92±9.17 | 80.97±8.5 | 81.32±6.29 | 84.55±12.36 | 0.77 | 0.82 | 0.79 | 1.02 | 0.46 | 0.73 |
| UA, mmol/L | 367.66±72.18 | 295.91±83.32 | 363.13±65.51 | 353.94±46.32 | 0.01 | 0.04 | 0.82 | 1.00 | 0.57 | 0.77 |
| FBG, mmol/L | 5.11±0.52 | 5.14±0.49 | 5.22±0.71 | 5.1±0.86 | 0.89 | 0.92 | 0.53 | 0.80 | 0.94 | 0.94 |
| CHOL, mmol/L | 5.05±0.83 | 4.51±1.25 | 5.19±0.92 | 5.6±1.92 | 0.11 | 0.2 | 0.55 | 0.81 | 0.18 | 0.34 |
| TG, mmol/L | 1.53±0.88 | 1.89±1.44 | 2.26±1 | 2.64±2.95 | 0.33 | 0.44 | 5.89×10^-3^ | 0.03 | 0.05 | 0.13 |
| HDL, mmol/L | 1.35±0.31 | 1.42±0.27 | 1.18±0.21 | 1.1±0.23 | 0.54 | 0.63 | 0.02 | 0.06 | 9.22×10^-3^ | 0.08 |
| LDL, mmol/L | 3.11±0.8 | 2.3±0.74 | 3.12±0.65 | 3.31±1.11 | 6.00×10^-3^ | 0.02 | 0.95 | 0.99 | 0.52 | 0.74 |
| FFA, mmol/L | 0.58±0.2 | 0.44±0.12 | 0.62±0.16 | 0.76±0.36 | 0.05 | 0.12 | 0.43 | 0.67 | 0.04 | 0.13 |

Data are mean ± SD. P values were adjusted with Bonferroni method. Education levels: illiteracy/primary school/middle school/college; Income levels: monthly 0~1000¥/1000~3000¥/3000~5000¥/5000~10000¥/10000+¥. HAM-A: Hamilton Rating Scale for Anxiety; HAM-D: Hamilton Depression Rating Scale; ALB: Albumin; GLB: Globulin; ALT: Alanine transaminase; AST: Aspartate aminotransferase; TBIL: Serum total bilirubin; PAB: Prealbumin; Cr: Creatinine; UA: [Uric](C:/Users/Euro/AppData/Local/youdao/dict/Application/8.9.9.0/resultui/html/index.html#/javascript:;) [Acid](C:/Users/Euro/AppData/Local/youdao/dict/Application/8.9.9.0/resultui/html/index.html#/javascript:;); FBG: ; CHOL: [cholesterol](C:/Users/Euro/AppData/Local/youdao/dict/Application/8.9.9.0/resultui/html/index.html#/javascript:;); TG: [triacylglycerol](C:/Users/Euro/AppData/Local/youdao/dict/Application/8.9.9.0/resultui/html/index.html#/javascript:;); HDL: [high-density lipoprotein](C:/Users/Euro/AppData/Local/youdao/dict/Application/8.9.9.0/resultui/html/index.html#/javascript:;); LDL: [low-density lipoprotein](C:/Users/Euro/AppData/Local/youdao/dict/Application/8.9.9.0/resultui/html/index.html#/javascript:;); FFA: [free](C:/Users/Euro/AppData/Local/youdao/dict/Application/8.9.9.0/resultui/html/index.html#/javascript:;) [fatty](C:/Users/Euro/AppData/Local/youdao/dict/Application/8.9.9.0/resultui/html/index.html#/javascript:;) [acid](C:/Users/Euro/AppData/Local/youdao/dict/Application/8.9.9.0/resultui/html/index.html#/javascript:;).

Table S2. Bile acid profiles of the three periods after METH withdrawal

| Bile Acid | HC(n=10) | T1 (n=9) | T2 (n=10) | T3 (n=10) |  | HC vs. M |  | HC vs. T1 | HC vs. T2 | HC vs. T3 |  | T1 vs. T2 vs. T3 |
| --- | --- | --- | --- | --- | --- | --- | --- | --- | --- | --- | --- | --- |
|  |  |  |  |  |  | *p_.adj_* |  | *p_.adj_* | *p_.adj_* | *p_.adj_* |  | *p* |
| Total BAs | 5826.48±762.96 | 3840.77±753.83 | 2450.49±386.98 | 4409.63±800.56 |  | 8.66×10-3 |  | 0.21 | 5.21×10-3 | 0.59 |  | 9.13×10-3 |
| TɑMCA | 1.8±0.92 | 1.37±0.91 | 4.25±0.93 | 2.48±1.02 |  | 0.43 |  | 0.64 | 0.08 | 0.63 |  | 0.31 |
| THCA | 4.74±0.26 | 5.41±2.2 | 1.35±0.71 | 4.25±1.38 |  | 0.05 |  | 1.00 | 0.03 | 1.00 |  | 0.04 |
| TCA | 11.35±2.04 | 12.28±5.44 | 5.26±2.66 | 19.94±9.13 |  | 0.86 |  | 0.85 | 0.09 | 0.65 |  | 0.25 |
| TUDCA | 6.61±2.89 | 4.73±1.28 | 0.65±0.31 | 4.09±1.57 |  | 0.22 |  | 0.58 | 0.24 | 0.46 |  | 0.63 |
| THDCA | 1.44±0.7 | 0.48±0.2 | 1.44±0.70 | 0.65±0.31 |  | 0.06 |  | 0.26 | 0.06 | 0.62 |  | 0.32 |
| TCDCA | 143.5±21.85 | 117.68±37.33 | 74.42±21 | 136.82±44.08 |  | 0.05 |  | 0.45 | 0.04 | 0.7 |  | 0.05 |
| TLCA | 0.23±0.18 | 0.45±0.33 | 0.93±0.42 | 0.86±0.43 |  | 0.21 |  | 0.55 | 0.14 | 0.19 |  | 0.67 |
| UCA | 2.79±0.46 | 3.24±0.7 | 1.54±0.36 | 2.83±0.77 |  | 0.06 |  | 1.00 | 2.61×10-3 | 1.00 |  | 0.02 |
| bCA | 5.69±2.22 | 1.51±0.76 | 1.3±0.66 | 3.11±1.48 |  | 0.05 |  | 0.21 | 0.07 | 0.34 |  | 0.43 |
| aMCA | 3.37±1.12 | 0.77±0.38 | 0.63±0.27 | 1.1±0.27 |  | 0.02 |  | 0.02 | 0.03 | 0.35 |  | 0.01 |
| bMCA | 3.68±0.9 | 3.03±0.79 | 2.37±0.62 | 1.67±0.47 |  | 0.14 |  | 0.55 | 0.35 | 0.07 |  | 0.45 |
| HCA | 47.53±14.11 | 22.59±2.25 | 17.54±0.89 | 23.02±3.06 |  | 0.01 |  | 0.06 | 3.05×10-3 | 0.05 |  | 4.18×10-3 |
| CA | 664.16±212.47 | 119±41.2 | 73.6±8.21 | 125.05±35.97 |  | 7.44×10-5 |  | 8.12×10-5 | 1.08×10-5 | 9.38×10-4 |  | 4.05×10-5 |
| NorCA | 8.8±1.61 | 5.5±1.54 | 2.42±0.71 | 5.59±1.49 |  | 0.01 |  | 0.623 | 2.25×10-3 | 0.573 |  | 5.25×10-3 |
| GHCA | 19.46±3.05 | 20.03±6.35 | 7.2±0.98 | 17.64±4.65 |  | 0.35 |  | 0.96 | 0.01 | 0.67 |  | 0.01 |
| GCA | 230.46±38.23 | 234.41±77.49 | 129.87±28.68 | 224.43±76.42 |  | 0.27 |  | 0.83 | 0.04 | 0.74 |  | 0.47 |
| GUDCA | 240.47±95.99 | 140.81±41.49 | 98.85±29.74 | 119.42±36.85 |  | 0.07 |  | 0.37 | 0.38 | 0.25 |  | 0.78 |
| GHDCA | 15.05±7.22 | 2.53±0.42 | 1.27±0.28 | 3.06±1.06 |  | 0.29 |  | 0.12 | 0.07 | 0.42 |  | 0.18 |
| GCDCA | 2419.59±356.8 | 1915.92±579.95 | 1033.79±261.43 | 2020.7±381.99 |  | 0.11 |  | 0.42 | 0.03 | 0.94 |  | 0.04 |
| GDCA | 164.87±59.73 | 167.98±77.95 | 123.18±31.45 | 256.76±124.79 |  | 0.77 |  | 0.98 | 0.35 | 0.42 |  | 0.62 |
| GLCA-3S | 3.87±0.73 | 4.67±1.13 | 7.91±1.68 | 6.81±2.05 |  | 0.13 |  | 0.99 | 0.03 | 0.78 |  | 0.38 |
| muroCA | 7.61±2.54 | 8.29±2.63 | 7.92±2.66 | 11.56±3.51 |  | 0.23 |  | 0.85 | 0.93 | 0.38 |  | 0.22 |
| bUDCA | 138.13±55.59 | 136.01±33.04 | 103.04±26.35 | 111.64±18.05 |  | 0.59 |  | 0.98 | 0.58 | 0.22 |  | 0.22 |
| bHDCA | 6.09±1.33 | 4.5±0.94 | 3.71±0.78 | 4.1±0.61 |  | 0.08 |  | 0.35 | 0.32 | 0.39 |  | 0.88 |
| UDCA | 144.09±39.83 | 66.37±12.45 | 54.55±12.35 | 89.71±25.93 |  | 0.02 |  | 0.81 | 4.13×10-3 | 1.00 |  | 0.13 |
| HDCA | 35.08±23.08 | 8.42±2.17 | 5.39±1.62 | 8.58±2.84 |  | 0.06 |  | 0.39 | 0.33 | 0.37 |  | 0.62 |
| bDCA | 27.5±7.41 | 23.4±6.57 | 36.89±7.64 | 54.5±19.86 |  | 0.23 |  | 0.69 | 0.39 | 0.33 |  | 0.37 |
| CDCA | 1074.17±193.75 | 353.75±138.51 | 252.17±58.88 | 674.18±322.98 |  | 9.79×10-3 |  | 0.01 | 4.27×10-3 | 0.19 |  | 3.12×10-3 |
| DCA | 214.81±68.46 | 131.52±51.27 | 136.12±38.1 | 347.6±158.38 |  | 0.96 |  | 0.36 | 0.33 | 0.46 |  | 0.24 |
| LCA | 3.85±0.56 | 4.67±1.28 | 8.03±2.04 | 5.97±1.33 |  | 0.26 |  | 0.66 | 0.07 | 0.27 |  | 0.36 |
| LCA_3S | 4.03±0.74 | 4.2±1.33 | 7.51±1.89 | 6.67±2.12 |  | 0.26 |  | 0.92 | 0.23 | 0.26 |  | 0.44 |
| dehydroLCA | 4.61±0.55 | 4.13±0.67 | 5.28±1.09 | 4.23±0.69 |  | 0.97 |  | 0.68 | 0.69 | 0.77 |  | 0.67 |
| 7_ketoLCA | 23.1±5.7 | 8.98±2.65 | 5.14±1.89 | 15.02±4.86 |  | 0.04 |  | 0.34 | 0.02 | 1.00 |  | 0.03 |
| 6_ketoLCA | 2.24±0.38 | 1.83±0.46 | 2.19±0.37 | 1.4±0.47 |  | 0.67 |  | 0.37 | 0.96 | 0.28 |  | 0.44 |
| 12_ketoLCA | 1.27±0.62 | 4.66±2.8 | 2.53±1.5 | 5.46±2.2 |  | 0.28 |  | 0.26 | 0.43 | 0.08 |  | 0.62 |
| apoCA | 10.52±3.12 | 7.05±1.82 | 11.08±5.48 | 5.15±1.98 |  | 0.30 |  | 0.66 | 0.96 | 0.26 |  | 0.32 |
| 6_7_diketoLCA | 1.96±0.06 | 1.69±0.22 | 1.34±0.29 | 1.39±0.31 |  | 0.08 |  | 0.24 | 0.08 | 0.09 |  | 0.64 |
| 7,12_diketoLCA | 0.84±0.25 | 1.83±1.01 | 0.89±0.49 | 0.36±0.13 |  | 0.82 |  | 0.66 | 0.94 | 0.22 |  | 0.27 |
| 7-DHCA | 3.04±1.4 | 3.01±0.59 | 2.78±1.27 | 2.06±0.56 |  | 0.72 |  | 0.98 | 0.89 | 0.32 |  | 0.74 |
| 12-DHCA | 1.59±0.49 | 0.97±0.35 | 2.1±0.85 | 1.83±0.65 |  | 0.92 |  | 0.66 | 0.62 | 0.76 |  | 0.48 |
| 3-DHCA | 1.41±0.7 | 0.59±0.35 | 0.2±0.13 | 0.73±0.46 |  | 0.09 |  | 0.66 | 0.22 | 0.46 |  | 0.36 |
| CDCA-24Gln | 121.09±48.09 | 280.49±92.24 | 211.09±91.45 | 77.17±31.63 |  | 0.46 |  | 0.26 | 0.43 | 0.46 |  | 0.28 |
| CA:CDCA | 0.56±0.09 | 0.4±0.07 | 0.42±0.07 | 0.32±0.06 |  | 0.33 |  | 0.47 | 0.421 | 0.10 |  | 0.14 |
| DCA:CA | 0.66±0.23 | 1.51±0.5 | 2.2±0.68 | 2.27±0.75 |  | 0.05 |  | 1.00 | 0.03 | 0.45 |  | 0.04 |
| TLCA:CDCA | 0 | 0 | 0.01±0 | 0 |  | 0.38 |  | 1.00 | 0.40 | 0.72 |  | 0.25 |
| LCA:CDCA | 0.01±0 | 0.04±0.02 | 0.06±0.02 | 0.02±0 |  | 0.02 |  | 0.3 | 7.21×10-3 | 0.24 |  | 0.01 |
| GDCA:DCA | 7.54±1.97 | 12.91±5.67 | 7.26±2.69 | 7.76±3.58 |  | 0.69 |  | 1.00 | 1.00 | 1.00 |  | 0.75 |

Data are mean ± SD. *P* values were adjusted with Bonferroni method. The abbreviations of each bile Acid were show in Table S3.

#### Table S3. Abbreviations of Bile Acids and Bile Acid Species.

| Abbreviation | Bile acid | Abbreviation | Bile acid |
| --- | --- | --- | --- |
| TɑMCA | Tauro-ɑ-muricholic acid | muroCA | Murocholic acid |
| THCA | Taurohyocholic acid | bUDCA | β-ursodeoxycholic Acid |
| TCA | Taurocholic acid | bHDCA | β-hyodeoxycholic acid |
| TUDCA | Tauroursodeoxycholic acid | UDCA | Ursodeoxycholic acid |
| THDCA | Taurohyodeoxycholic acid | HDCA | Hyodeoxycholic acid |
| TCDCA | Taurochenodeoxycholic acid | bDCA | β-deoxycholic acid |
| TLCA | Taurolithocholic acid | CDCA | Chenodeoxycholic acid |
| UCA | Ursocholic acid | DCA | Deoxycholic acid |
| bCA | 3β-cholic acid | LCA | Lithocholic acid |
| αMCA | α-muricholic acid | LCA_3S | Lithocholic acid 3-sulfate |
| βMCA | β-muricholic acid | dehydroLCA | Dehydrolithocholic acid |
| HCA | Hyocholic acid | 7_ketoLCA | 7-ketolithocholic acid |
| CA | Cholic acid | 6_ketoLCA | 6-ketolithocholic acid |
| NorCA | 23-norcholic acid | 12_ketoLCA | 12-ketolithocholic acid |
| GHCA | Glycohyocholic acid | apoCA | apocholic acid |
| GCA | Glycocholic acid | 6_7_diketoLCA | 6,7-diketolithocholic acid |
| GUDCA | Glycoursodeoxycholic acid | 7,12_diketoLCA | 7,12-diketolithocholic acid |
| GHDCA | Glycohyodeoxycholic acid | 7-DHCA | 7-dehydrocholic acid |
| GCDCA | Glycochenodeoxycholic acid | 12-DHCA | 12-dehydrocholic acid |
| GDCA | Glycodeoxycholic acid | 3-DHCA | 3-dehydrocholic acid |
| GLCA-3S | Glycolithocholic acid | CDCA-24Gln | Chenodeoxycholic acid 24-acyl β-D-glucuronide |
