# Supplementary figures and images for: Psychiatric Comorbidities and Liver Injury Are Associated With Unbalanced Plasma Bile Acid Profile During Methamphetamine Withdrawal
Source: Front Endocrinol (Lausanne). 2022 Jan 3;12:801686. doi: 10.3389/fendo.2021.801686 (PMC8761939; doi:10.3389/fendo.2021.801686)

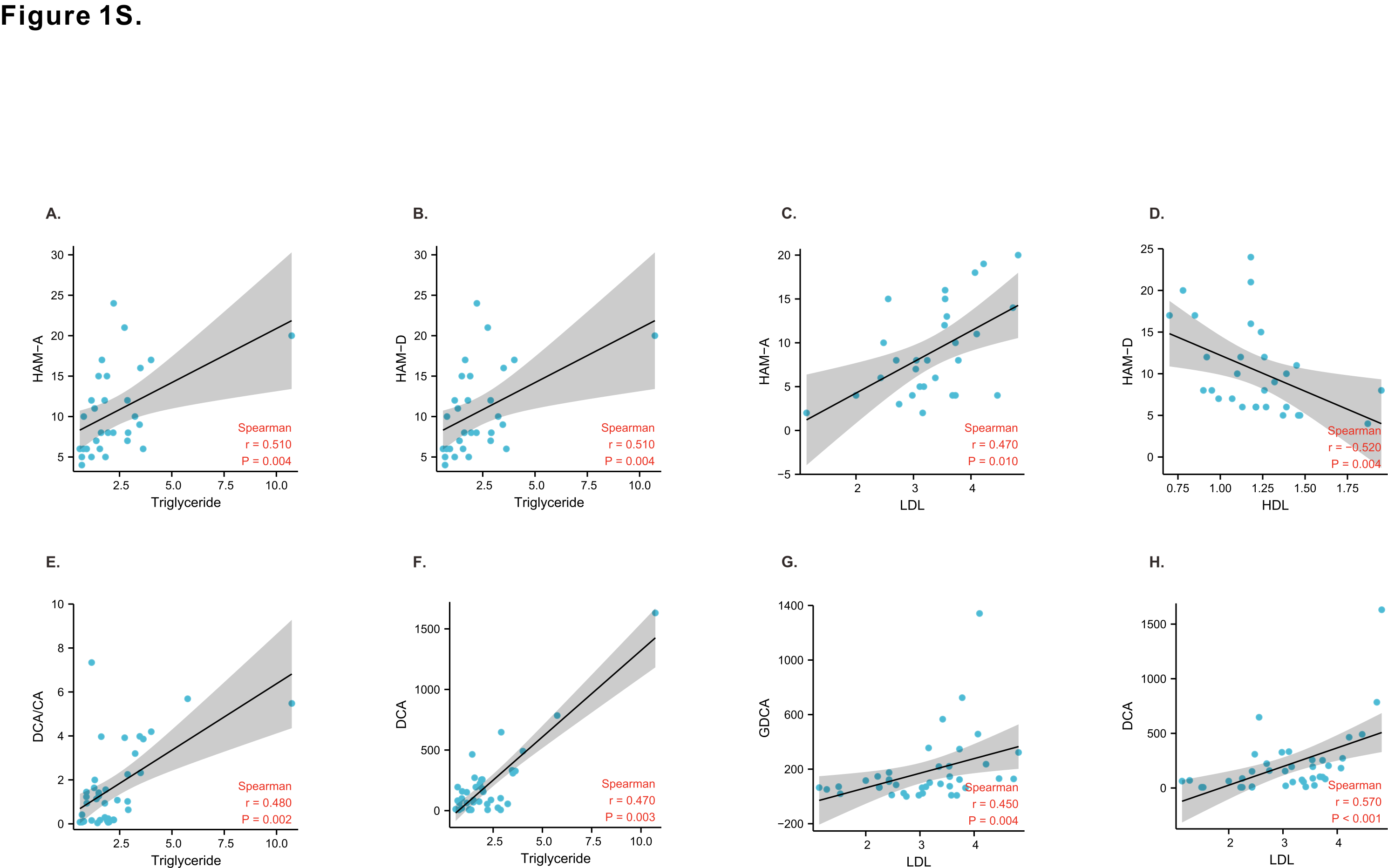

Supplement: Supplementary Figure 1 — Associations of dyslipidemia parameters with psychiatric comorbidities (A–D) and bile acid concentrations (E–H) after METH withdrawal, as determined by Spearman correlation analysis. [file DataSheet_1.zip › 2Revised Supplementary Material Presentation/Figure1S.tif]
